# A Human Activity Recognition System Using Skeleton Data from RGBD Sensors

**DOI:** 10.1155/2016/4351435

**Published:** 2016-03-16

**Authors:** Enea Cippitelli, Samuele Gasparrini, Ennio Gambi, Susanna Spinsante

**Affiliations:** Dipartimento di Ingegneria dell'Informazione, Università Politecnica delle Marche, 60131 Ancona, Italy

## Abstract

The aim of Active and Assisted Living is to develop tools to promote the ageing in place of elderly people, and human activity recognition algorithms can help to monitor aged people in home environments. Different types of sensors can be used to address this task and the RGBD sensors, especially the ones used for gaming, are cost-effective and provide much information about the environment. This work aims to propose an activity recognition algorithm exploiting skeleton data extracted by RGBD sensors. The system is based on the extraction of key poses to compose a feature vector, and a multiclass Support Vector Machine to perform classification. Computation and association of key poses are carried out using a clustering algorithm, without the need of a learning algorithm. The proposed approach is evaluated on five publicly available datasets for activity recognition, showing promising results especially when applied for the recognition of AAL related actions. Finally, the current applicability of this solution in AAL scenarios and the future improvements needed are discussed.

## 1. Introduction

People ageing is one of the main problems in modern and developed society and Active and Assisted Living (AAL) tools may allow to reduce social costs by helping older people to age at home. In the last years, several tools have been proposed to improve quality of life of elderly people, from the remote control of health conditions to the improvement of safety [1].

Human activity recognition (HAR) is a hot research topic since it may enable different applications, from the most commercial (gaming or Human Computer Interaction) to the most assistive ones. In this area, HAR can be applied, for example, to detect dangerous events or to monitor people living alone. This task can be accomplished using different sensors, mainly represented by wearable sensors or vision-based devices [2], even if there is a growing number of researchers working on radio-based solutions [3], or others who fuse data captured from wearable and ambient sensors [4, 5]. The availability in the market of RGBD sensors fostered the development of promising approaches to build reliable and cost-effective solutions [6]. Using depth sensors, like Microsoft Kinect or other similar devices, it is possible to design activity recognition systems exploiting depth maps, which are a good source of information because they are not affected by environment light variations, can provide body shape, and simplify the problem of human detection and segmentation [7]. Furthermore, the availability of skeleton joints extracted from the depth frames allows having a compact representation of the human body that can be used in many applications [8]. HAR may have strong privacy-related implications, and privacy is a key factor in AAL. RGBD sensors are much more privacy preserving than traditional video cameras: thanks to the easy computation of the human silhouette, it is possible to achieve an even higher level of privacy by using only the skeleton to represent a person [9].

In this work, a human action recognition algorithm exploiting the skeleton provided by Microsoft Kinect is proposed, and its application in AAL scenarios is discussed. The algorithm starts from a skeleton model and computes posture features. Then, a clustering algorithm selects the key poses, which are the most informative postures, and a vector containing the activity features is composed. Finally, a multiclass Support Vector Machine (SVM) is exploited to obtain different activities. The proposed algorithm has been tested on five publicly available datasets and it outperforms the state-of-the-art results obtained from two of them, showing interesting performances in the evaluation of activities specifically related to AAL.

The paper is organized as follows: Section 2 reviews related works in human activity recognition using RGBD sensors; Section 3 describes the proposed algorithm, whereas the experimental results are shown in Section 4; Section 5 discusses the applicability of this system to the AAL scenario; and finally Section 6 provides concluding remarks.

## 2. Related Works

In the last years, many solutions for human activity recognition have been proposed, some of them aimed to extract features from depth data, such as [10], where the main idea is to evaluate spatiotemporal depth subvolume descriptors. A group of hypersurface normals (polynormal), containing geometry and local motion information, is extracted from depth sequences. The polynormals are then aggregated to constitute the final representation of the depth map, called Super Normal Vector (SNV). This representation can include also skeleton joint trajectories, improving the recognition results when people move a lot in a sequence of depth frames. Depth images can be seen as sequence features modeled temporally as subspaces lying on the Grassmann manifold [11]. This representation, starting from the orientation of the normal vector at every surface point, describes the geometric appearance and the dynamic of human body without using joint position. Other works proposed holistic descriptors: the HON4D descriptor [12], which is based on the orientations of normal surfaces in 4D, and HOPC descriptor [13], which is able to represent the geometric characteristics of a sequence of 3D points.

Other works exploit both depth and skeleton data; for example, the* 3.5D representation* combines the skeleton joint information with features extracted from depth images, in the region surrounding each node of interest [14]. The features are extracted using an extended Independent Subspace Analysis (ISA) algorithm by applying it only to local region of joints instead of the entire video, thus improving the training efficiency. The depth information makes it easy to extract the human silhouette, which can be concatenated with normalized skeleton features, to improve the recognition rate [15]. Depth and skeleton features can be combined at different levels of the activity recognition algorithm. Althloothi et al. [16] proposed a method where the data are fused at the kernel level, instead of the feature level, using the Multiple Kernel Learning (MKL) technique. On the other hand, fusion at the feature level of spatiotemporal features and skeleton joints is performed in [17]. In such a work, several spatiotemporal interest point detectors, such as Harris 3D, ESURF [18], and HOG3D [19], have been fused using regression forests with the skeleton joint features consisting of posture, movement, and offset information.

Skeleton joints extracted from depth frames can be combined also with RGB data. Luo et al. [20] proposed a human action recognition framework where the pairwise relative positions of joints and Center-Symmetric Motion Local Ternary Pattern (CS-Mltp) features from RGB are fused both at feature level and at classifier level. Spatiotemporal Interest Points (STIP) are typically used in activity recognition where data are represented by RGB frames. This approach can be also extended to depth and skeleton data, combining the features with random forests [21]. The results are very good, but depth estimation noise and background may have an impact on interest point detection, so the depth STIP features have to be constrained using skeleton joint positions, or RGB videos. Instead of using spatiotemporal features, another approach for human activity recognition relies on graph-based methods for sequential modeling of RGB data. This concept can be extended to depth information, and an approach based on coupled Hidden Conditional Random Fields (cHCRF) model, where visual feature sequences are extracted from RGB and depth data, has been proposed [22]. The main advantage of this approach is the capability to preserve the dynamics of individual sequences, even if the complementary information from RGB and depth are shared.

Some previous works simply rely on Kinect skeleton data, such as the proposed algorithm. Maybe they are simpler approaches but in many cases they can achieve performance very close to the algorithms exploiting multimodal data, and sometimes they also perform better than those solutions. Devanne et al. [23] proposed representing human actions by spatiotemporal motion trajectories in a 60-dimensional space, since they considered 20 joints, each of them with 3 coordinates. Then, an elastic metric, which means a metric invariant to speed and time of the action, within a Riemannian shape space, is employed to represent the distance between two curves. Finally, the action recognition problem can be seen as a classification in the Riemannian space, using a *k*-Nearest-Neighbor (*k*-NN) classifier. Other skeleton representations have been proposed. The APJ3D representation [24] is constituted starting by a subset of 15 skeleton joints, from which the relative positions and local spherical angles are computed. After a selection of key-postures, the action is partitioned using a reviewed Fourier Temporal Pyramid [25] and the classification is made by random forests. Another joint representation is called HOJ3D [26], where the 3D space is partitioned into *n* bins and the joints are associated with each bin using a Gaussian weight function. Then, a discrete Hidden Markov Model (HMM) is employed to model the temporal evolution of the postures, attained using a clustering algorithm. A human action can be characterized also by a combination of static posture features, representing the actual frame, consecutive motion features, computed using the actual and the previous frames, and overall dynamics features, which consider the actual and the initial frames [27]. Taha et al. [28] also exploit joints spherical coordinates to represent the skeleton and a framework composed of a multiclass SVM and a discrete HMM to recognize activities constituted by many actions. Other approaches exploit a double machine learning algorithm to classify actions; for example, Gaglio et al. [29] consider a multiclass SVM to estimate the postures and a discrete HMM to model an activity as a sequence of postures. Also in [30], human actions are considered as a sequence of body poses over time, and skeletal data are processed to obtain invariant pose representations, given by 8 pairs of angles. Then the recognition is realized using the representation in the dissimilarity space, where different feature trajectories maintain discriminant information and have a fixed-length representation. Ding et al. [31] proposed a Spatiotemporal Feature Chain (STFC) to represent the human actions by trajectories of joint positions. Before using the STFC model, a graph is used to erase periodic sequences, making the solution more robust to noise and periodic sequence misalignment. Slama et al. [32] exploited the geometric structure of the Grassmann manifold for action analysis. In fact, considering the problem as a sequence matching task, this manifold allows considering an action sequence as a point on its space and provides tools to make statistical analysis. Considering that the relative geometry between body parts is more meaningful than their absolute locations, rotations and translations required to perform rigid body transformations can be represented as points in a Special Euclidean, SE(3), group. Each skeleton can be represented as a point in the Lie group SE(3) × SE(3)×⋯×SE(3), and a human action can be modeled as a curve in this Lie group [33]. The same skeleton feature is also used in [34], where Manifold Functional PCA (mfPCA) is employed to reduce feature dimensionality. Some works developed techniques to automatically select the most informative joints, aiming at increasing the recognition accuracy and reducing the noise effect on the skeleton estimation [35, 36].

The work presented in this paper is based on the concept that an action can be seen as a sequence of informative postures, which are known as “key poses.” This idea has been introduced in [37] and used in other subsequent proposals [15, 36, 38]. While in previous works, a fixed set of key poses (*K*) is extracted for each action of the dataset, considering all the training sequences, in this work, the clustering algorithm which selects the most informative postures is executed for each sequence, thus selecting a different set of *K* poses which constitutes the feature vector. This procedure avoids the application of a learning algorithm which has to find the nearest neighbor key pose for each frame constituting the sequence.

## 3. Activity Recognition Algorithm

**Figure 1:**
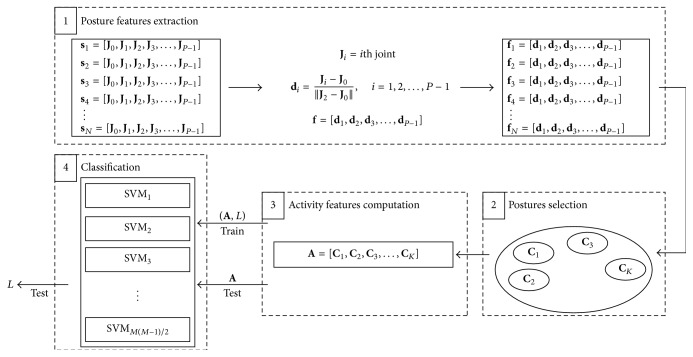
The general scheme of the activity recognition algorithm is composed of 4 steps. In the first step, the posture feature vectors are computed for each skeleton frame; then the postures are selected and an activity features vector is created. Finally, a multiclass SVM is exploited for classification.

The proposed algorithm for activity recognition starts from skeleton joints and computes a vector of features for each activity. Then, a multiclass machine learning algorithm, where each class represents a different activity, is exploited for classification purpose. Four main steps constitute the whole algorithm; they are represented in Figure 1 and are discussed in the following:

1. *Posture Features Extraction*. The coordinates of the skeleton joints are used to evaluate the feature vectors which represent human postures.
2. *Postures Selection*. The most important postures for each activity are selected.
3. *Activity Features Computation*. A feature vector representing the whole activity is created and used for classification.
4. *Classification*. The classification is realized using a multiclass SVM implemented with the “one-versus-one” approach.

First, there is the need to extract the features from skeleton data representing the input to the algorithm. The joint extraction algorithm proposed in [8] is included in the Kinect libraries and is ready to use. The choice to consider Kinect skeleton is motivated by the fact that it is easy to have a compact representation of the human body. Starting from skeleton data, many features which are able to represent a human pose, and consequently a human action, have been proposed in the literature. In [6], the authors found that many features can be computed from skeleton joints. The simplest features can be extracted by joint locations or from their distances, considering spatial information. Other features may involve joints orientation or motion, considering spatial and temporal data. More complex features may be initially based on the estimation of a plane considering some joints. Then by measuring the distances between this plane and other joints, the features can be extracted.

The proposed algorithm exploits spatial features computed from 3D skeleton coordinates, without including the time information in the computation, in order to make the system independent of the speed of movement. The feature extraction method has been introduced in [36] and it is here adopted with small differences. For each skeleton frame, a posture feature vector is computed. Each joint is represented by **J**_*i*_, a three-dimensional vector in the coordinate space of Kinect. The person can be found at any place within the coverage area of Kinect, and the coordinates of the same joint may assume different values. It is necessary to compensate this effect, by using a proper features computation algorithm. A straightforward solution is to compensate the position of the skeleton by centering the coordinate space in one skeleton joint. Considering a skeleton composed of *P* joints, **J**_0_ being the coordinates of the torso joint, and **J**_2_ being the coordinates of the neck joint, the *i*th joint feature **d**_*i*_ is the distance vector between **J**_*i*_ and **J**_0_, normalized to the distance between **J**_2_ and **J**_0_:

(1) $$\begin{array}{c} d_{i}=\frac{J_{i}-J_{0}}{\left\|J_{2}-J_{0}\right\|},\;i=1,2,\ldots ,P-1. \end{array}$$

This feature is invariant to the position of the skeleton within the coverage area of Kinect; furthermore, the invariance of the feature to the build of the person is obtained by normalization with respect to the distance between the neck and torso joints. These features may be seen as a set of distance vectors which connect each joint to the joint of the torso. A posture feature vector **f** is created for each skeleton frame:

(2) $$\begin{array}{c} f=\left[\;d_{1},d_{2},d_{3},\ldots ,d_{P-1}\;\right]. \end{array}$$

A set of *N* feature vectors is computed, having an activity constituted by *N* frames.

The second phase concerns the human postures selection, with the aim of reducing the complexity and increasing generality by representing the activity by means of only a subset of poses, without using all the frames. A clustering algorithm is used to process *N* feature vectors constituting the activity, by grouping them into *K* clusters. The well-known *k*-means clustering algorithm, based on the squared Euclidean distance as a metric, can be used to group together the frames representing similar postures. Considering an activity composed of *N* feature vectors [**f**_1_, **f**_2_, **f**_3_,…, **f**_*N*_], the *k*-means algorithm gives as outputs *N* clusters ID (one for each feature vector) and *K* vectors [**C**_1_, **C**_2_, **C**_3_,…, **C**_*K*_] that represent the centroids of each cluster. The feature vectors are partitioned into clusters *S*_1_, *S*_2_,…, *S*_*K*_ so as to satisfy the condition expressed by

(3) $$\begin{array}{c} arg\;\underset{S}{min}\overset{K}{\underset{j=1}{\sum }}\;\underset{f_{i}\in S_{j}}{\sum }{\left\|\;f_{i}-C_{j}\;\right\|}^{2}. \end{array}$$

The *K* centroids can be seen as the main postures, which are the most important feature vectors. Unlike classical approaches based on key poses, where the most informative postures are evaluated by considering all the sequences of each activity, in the proposed solution the clustering algorithm is executed for each sequence. This avoids the application of a learning algorithm required to associate each frame to the closest key pose and allows to have a more compact representation of the activity.

The third phase is related to the computation of a feature vector which models the whole activity, starting from the *K* centroid vectors computed by the clustering algorithm. In more detail, [**C**_1_, **C**_2_, **C**_3_,…, **C**_*K*_] vectors are sorted considering the order in which the cluster's elements occur during the activity. The activity features vector is composed of concatenating the sorted centroid vectors. For example, considering an activity featured by *N* = 10 and *K* = 4, after running the *k*-means algorithm, one of the possible outputs could be the following sequence of cluster IDs: [2,2, 2,3, 3,1, 1,4, 4,4], meaning that the first three posture vectors belong to cluster 2, the fourth and the fifth are associated with cluster 3, and so on. In this case, the activity features vector is **A** = [**C**_2_, **C**_3_, **C**_1_, **C**_4_]. A feature activity vector has a dimension of 3*K*(*P* − 1) that can be handled without using dimensionality reduction algorithms, such as PCA, if *K* is small.

The classification step aims to associate each feature activity vector to the correct activity. Many machine learning algorithms may be applied to fulfil this task, among them a SVM. Considering a number of *l* training vectors **x**_*i*_ ∈ *R* ^*n*^ and a vector of labels *y* ∈ *R* ^*l*^, where *y*_*i*_ ∈ {−1,1}, a binary SVM can be formulated as follows [39]:

(4) minw,b,ξ 12wTw+C∑i=1lξisubject  to yiwTϕxi+b≥1−ξi, ξi≥0,  i=1,…,l,

where

(5) $$\begin{array}{c} w^{T}\phi \left(\;x\;\right)+b=0 \end{array}$$

is the optimal hyperplane that allows separation between classes in the feature space, *C* is a constant, and *ξ*_*i*_ are nonnegative variables which consider training errors. The function *ϕ* allows transforming between the features space and an higher dimensional space where the data are separable. Considering two training vectors **x**_*i*_ and **x**_*j*_, the kernel function can be defined as

(6) $$\begin{array}{c} K\left(\;x_{i},x_{j}\;\right)=\phi {\left(\;x_{i}\;\right)}^{T}\phi \left(\;x_{j}\;\right). \end{array}$$

In this work the Radial Basis Function (RBF) kernel has been used where

(7) $$\begin{array}{c} K\left(\;x_{i},x_{j}\;\right)=e^{-\gamma {\left\|x_{i}-x_{j}\right\|}^{2}},\;\gamma >0. \end{array}$$

It follows that *C* and *γ* are the parameters that have to be estimated prior using the SVM.

The idea herein exploited is to use a multiclass SVM, where each class represents an activity of the dataset. In order to extend the role from a binary to a multiclass classifier, some approaches have been proposed in the literature. In [40], the authors compared many methods and found that the “one-against-one” is one of the most suitable for practical use. It is implemented in LIBSVM [41] and it is the approach used in this work. The “one-against-one” approach is based on the construction of several binary SVM classifiers; in more detail, a number of *M*(*M* − 1)/2 binary SVMs are necessary in a *M*-classes dataset. This happens because each SVM is trained to distinguish between 2 classes, and the final decision is taken exploiting a voting strategy among all the binary classifiers. During the training phase, the activity feature vectors are given as inputs to the multiclass SVM, together with the label *L* of the action. In the test phase, the activity label is obtained from the classifier.

## 4. Experimental Results

**Figure 2:**
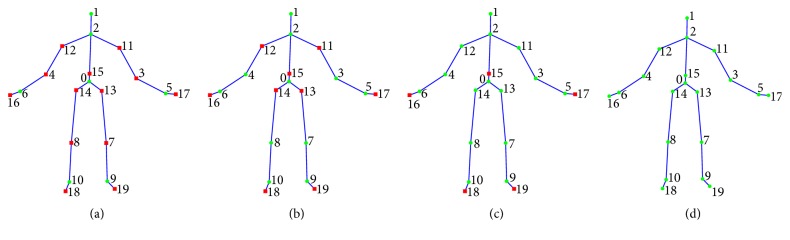
Subsets of joints considered in the evaluation of the proposed algorithm. The whole skeleton is represented by 20 joints, the selected ones are depicted as green circles, while the discarded joints are represented by red squares. Subsets of 7 (a), 11 (b), and 15 (c) joints, and the whole set of 20 joints (d), are selected.

The algorithm performance is evaluated on five publicly available datasets. In order to perform an objective comparison to previous works, the reference test procedures have been considered for each dataset. The performance indicators are evaluated using four different subsets of joints, shown in Figure 2, going from a minimum of 7 up to a maximum of 20 joints. Finally, in order to evaluate the performance in AAL scenarios, some suitable activities related to AAL are selected from the datasets, and the recognition accuracies are evaluated.

### 4.1. Datasets

Five different 3D datasets have been considered in this work, each of them including a different set of activities and gestures.

KARD dataset [29] is composed of 18 activities that can be divided into 10 gestures (*horizontal arm wave*,* high arm wave*,* two-hand wave*,* high throw*,* draw X*,* draw tick*,* forward kick*,* side kick*,* bend*, and* hand clap*), and eight actions (*catch cap*,* toss paper*,* take umbrella*,* walk*,* phone call*,* drink*,* sit down*, and* stand up*). This dataset has been captured in a controlled environment, that is, an office with a static background, and a Kinect device placed at a distance of 2-3 m from the subject. Some objects were present in the area, useful to perform some of the actions: a desk with a phone, a coat rack, and a waste bin. The activities have been performed by 10 young people (nine males and one female), aged from 20 to 30 years, and from 150 to 185 cm tall. Each person repeated each activity 3 times, creating a number of 540 sequences. The dataset is composed of RGB and depth frames captured at a rate of 30 fps, with a 640 × 480 resolution. In addition, 15 joints of the skeleton in world and screen coordinates are provided. The skeleton has been captured using OpenNI libraries [42].

The Cornell Activity Dataset (CAD-60) [43] is made by 12 different activities, typical of indoor environments. The activities are* rinsing mouth*,* brushing teeth*,* wearing contact lens*,* talking on the phone*,* drinking water*,* opening pill container*,* cooking-chopping*,* cooking-stirring*,* talking on couch*,* relaxing on couch*,* writing on whiteboard*, and* working on computer* and are performed in 5 different environments:* bathroom*,* bedroom*,* kitchen*,* living room*, and* office*. All the activities are performed by 4 different people: two males and two females, one of which is left-handed. No instructions were given to actors about how to perform the activities, the authors simply ensured that the skeleton was correctly detected by Kinect. The dataset is composed of RGB, depth, and skeleton data, with 15 joints available.

The UTKinect dataset [26] is composed of 10 different subjects (9 males and 1 female) performing 10 activities twice. The following activities are part of the dataset:* walk*,* sit down*,* stand up*,* pick up*,* carry*,* throw*,* push*,* pull*,* wave*, and* clap hands*. A number of 199 sequences are available because one sequence is not labeled, and the length of sample actions ranges from 5 to 120 frames. The dataset provides 640 × 480 RGB frames, and 320 × 240 depth frames, together with 20 skeleton joints, captured using Kinect for Windows SDK Beta Version, with a final frame rate of about 15 fps.

The Florence3D dataset [44] includes 9 different activities:* wave*,* drink from a bottle*,* answer phone*,* clap*,* tight lace*,* sit down*,* stand up*,* read watch*, and* bow*. These activities were performed by 10 different subjects, for 2 or 3 times, resulting in a total number of 215 sequences. This is a challenging dataset, since the same action is performed with both hands and because of the presence of very similar actions such as* drink from a bottle* and* answer phone*. The activities were recorded in different environments, and only RGB videos and 15 skeleton joints are available.

Finally, the MSR Action3D [45] represents one of the most used datasets for HAR. It includes 20 activities performed by 10 subjects, 2 or 3 times. In total, 567 sequences of depth (320 × 240) and skeleton frames are provided, but 10 of them have to be discarded because the skeletons are either missing or affected by too many errors. The following activities are included in the dataset:* high arm wave*,* horizontal arm wave*,* hammer*,* hand catch*,* forward punch*,* high throw*,* draw X*,* draw tick*,* draw circle*,* hand clap*,* two-hand wave*,* side boxing*,* bend*,* forward kick*,* side kick*,* jogging*,* tennis swing*,* tennis serve*,* golf swing*, and* pickup and throw*. The dataset has been collected using a structured-light depth camera at 15 fps; RGB data are not available.

### 4.2. Tests and Results

The proposed algorithm has been tested over the datasets detailed above, following the recommendations provided in each reference paper, in order to ensure a fair comparison to previous works. Following this comparison, a more AAL oriented evaluation has been conducted, which consists of considering only suitable actions for each dataset, excluding the gestures. Another type of evaluation regards the subset of skeleton joints that has to be included in the feature computation.

#### 4.2.1. KARD Dataset

Gaglio et al. [29] collected the KARD dataset and proposed some evaluation experiments on it. They considered three different experiments and two modalities of dataset splitting. The experiments are as follows:

- Experiment A: one-third of the data is considered for training and the rest for testing.
- Experiment B: two-thirds of the data is considered for training and the rest for testing.
- Experiment C: half of the data is considered for training and the rest for testing.

The activities constituting the dataset are split in the following groups:

- Gestures and Actions.
- Activity Set 1, Activity Set 2, and Activity Set 3, as listed in Table 1. Activity Set 1 is the simplest one since it is composed of quite different activities while the other two sets include more similar actions and gestures.

Each experiment has been repeated 10 times, randomly splitting training and testing data. Finally, the “new-person” scenario is also performed, that is, a leave-one-actor-out setting, consisting of training the system on nine of the ten people of the dataset and testing on the tenth. In the “new-person” test, no recommendation is provided about how to split the dataset, so we assumed that the whole dataset of 18 activities is considered. The only parameter that can be set in the proposed algorithm is the number of clusters *K*, and different subsets of skeleton joints are considered. Since only 15 skeleton joints are available, the fourth group of 20 joints (Figure 2(d)) cannot be considered.

For each test conducted on KARD dataset, the sequence of clusters *K* = [3,5, 10,15,20,25,30,35] has been considered. The results concerning Activity Set tests are reported in Table 2; it is shown that the proposed algorithm outperforms the originally proposed one in almost all the tests. The *K* parameter which gives the maximum accuracy is quite high, since it is 30 or 35 in most of the cases. It means that, for the KARD dataset, it is better to have a significant number of clusters representing each activity. This is possible also because the number of frames that constitutes each activity goes from a minimum of 42 in a sequence of* hand clap* gesture to a maximum of 310 for a* walk* sequence. Experiment A in Activity Set 1 shows the highest difference between the minimum and the maximum accuracy when varying the number of clusters. For example, considering *P* = 7, the maximum accuracy (98.2%) is shown in Table 2 and it is obtained with *K* = 35. The minimum accuracy is 91.4% and it is obtained with *K* = 5. The difference is quite high (6.8%) but this gap is reduced by considering more training data. In fact, Experiment B shows a difference of 0.5% in Activity Set 1, 1.1% in Activity Set 2, and 2.6% in Activity Set 3.

Considering the number of selected joints, the observation of Tables 2 and 3 lets us conclude that not all the joints are necessary to achieve good recognition results. In more detail, from Table 2, it can be noticed that the Activity Set 1 and Activity Set 2, which are the simplest ones, have good recognition results using a subset composed of *P* = 7 joints. The Activity Set 3, composed of more similar activities, is better recognized with *P* = 11 joints. In any case, it is not necessary to consider all the skeleton joints provided from KARD dataset.

**Figure 3:**
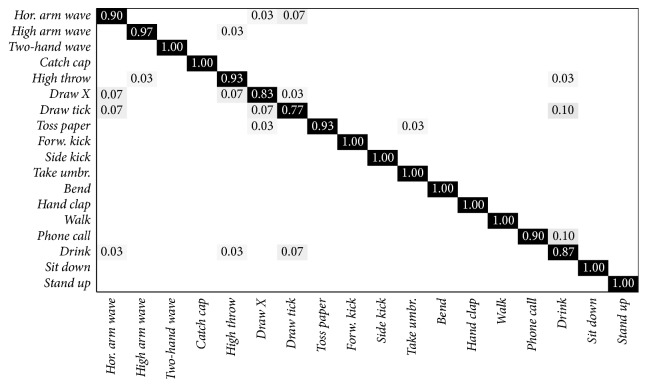
Confusion matrix of the “new-person” test on the whole KARD dataset.

The results obtained with the “new-person” scenario are shown in Table 4. The best result is obtained with *K* = 30, using a number of *P* = 11 joints. The overall precision and recall are about 10% higher than the previous approach which uses the KARD dataset. In this condition, the confusion matrix obtained is shown in Figure 3. It can be noticed that the actions are distinguished very well, only* phone call* and* drink* show a recognition accuracy equal or lower than 90%, and sometimes they are mixed with each other. The most critical activities are the* draw X* and* draw tick* gestures, which are quite similar.

From the AAL point of view, only some activities are relevant.  Table 3 shows that the eight actions constituting the KARD dataset are recognized with an accuracy greater than 98%, even if there are some similar actions such as* sit down* and* stand up*, or* phone call* and* drink*. Considering only the Actions subset, the lower recognition accuracy is 94.9% and it is obtained with *K* = 5 and *P* = 7. It means that the algorithm is able to reach a high recognition rate even if the feature vector is limited to 90 elements.

#### 4.2.2. CAD-60 Dataset

The CAD-60 dataset is a challenging dataset consisting of 12 activities performed by 4 people in 5 different environments. The dataset is usually evaluated by splitting the activities according to the environment; the global performance of the algorithm is given by the average precision and recall among all the environments. Two different settings were experimented for CAD-60 in [43]. The former is defined “new-person” and the latter is the so-called “have-seen.” “New-person” setting has been considered in all the works using CAD-60, so it is the one selected also in this work.

The most challenging element of the dataset is the presence of a left-handed actor. In order to increase the performance, which are particularly affected by this unbalancing in the “new-person” test, mirrored copies of each action are created, as suggested in [43]. For each actor, a left-handed and a right-handed version of each action are made available. The dummy version of the activity has been obtained by mirroring the skeleton with respect to the virtual sagittal plane that cuts the person in a half. The proposed algorithm is evaluated using three different sets of joints, from *P* = 7 to *P* = 15, and the sequence of clusters *K* = [3,5, 10,15,20,25,30,35], as in the KARD dataset.

The best results are obtained with the configurations *P* = 11 and *K* = 25, and the performance in terms of precision and recall, for each activity, is shown in Table 5. Very good results are given in* office* environment, where the average precision and recall are 96.4% and 95.8%, respectively. In fact, the activities of this environment are quite different, only* talking on phone* and* drinking water* are similar. On the other hand, the* living room* environment includes* talking on couch* and* relaxing on couch* in addition to* talking on phone* and* drinking water*, and it is the most challenging case, since the average precision and recall are 91% and 90.6%.

The proposed algorithm is compared to other works using the same “new-person” setting, and the results are shown in Table 6, which tells that the *P* = 11 configuration outperforms the state-of-the-art results in terms of precision, and it is only 1% lower in terms of recall. Shan and Akella [46] achieve very good results using a multiclass SVM scheme with a linear kernel. However, they train and test mirrored actions separately and then merge the results when computing average precision and recall. Our approach simply considers two copies of the same action given as input to the multiclass SVM and retrieves the classification results.

The reduced number of joints does not affect too much the average performance of the algorithm that reaches a precision of 92.7%, and a recall of 91.5%, with *P* = 7. Using all the available jonts, on the other hand, brings to a more substantial reduction of the performance, showing 87.9% and 86.7% for precision and recall, respectively, with *P* = 15. The best results for the proposed algorithm were always obtained with a high number of clusters (25 or 30). The reduction of this number affects the performance; for example, considering the *P* = 11 subset, the worst performance is obtained with *K* = 5 and with a precision of 86.6% and a recall of 86.0%.

From the AAL point of view, this dataset is composed only of actions, not gestures, so the dataset does not have to be separated to evaluate the performance in a scenario which is close to AAL.

#### 4.2.3. UTKinect Dataset

The UTKinect dataset is composed of 10 activities, performed twice by 10 subjects, and the evaluation setting proposed in [26] is the leave-one-out-cross-validation (LOOCV), which means that the system is trained on all the sequences except one and that one is used for testing. Each training/testing procedure is repeated 20 times, to reduce the random effect of *k*-means. For this dataset, all the different subsets of joints shown in Figure 2 are considered, since the skeleton is captured using Microsoft SDK which provides 20 joints. The considered sequence of clusters is only *K* = [3,4, 5], because the minimum number of frames constituting an action sequence is 5.

The results, compared with previous works, are shown in Table 7. The best results for the proposed algorithm are obtained with *P* = 7, which is the smallest set of joints considered. The result corresponds to a number of *K* = 4 clusters, but the difference with the other clusters is very low, only 0.6% with *K* = 5, that provided the worst result. The selection of different sets of joints, from *P* = 7 to *P* = 15, changes the accuracy only by a 2%, from 93.1% to 95.1%. In this dataset, the main limitation to the performance is given by the reduced number of frames that constitute some sequences and limits the number of clusters representing the actions. Vemulapalli et al. [33] reach the highest accuracy, but the approach is much more complex: after modeling skeleton joints in a Lie group, the processing scheme includes Dynamic Time Warping to perform temporal alignments and a specific representation called Fourier Temporal Pyramid before classification with one-versus-all multiclass SVM.

**Figure 4:**
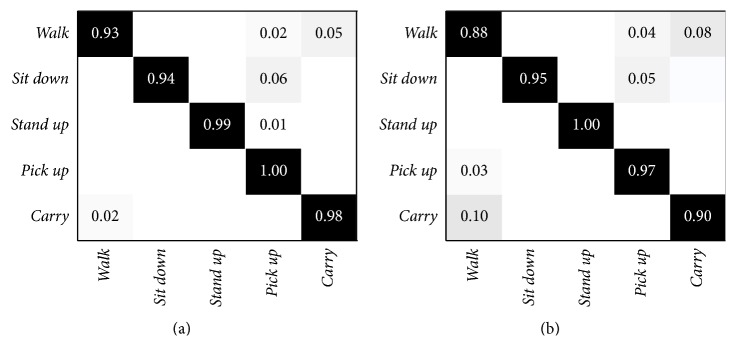
Confusion matrices of the UTKinect dataset with only AAL related activities. (a) Best accuracy confusion matrix, obtained with *P* = 7. (b) Worst accuracy confusion matrix, obtained with *P* = 15.

Contextualizing the UTKinect dataset to AAL involves the consideration of a subset of activities, which includes only actions and discards gestures. In more detail, the following 5 activities have been selected:* walk*,* sit down*,* stand up*,* pick up*, and* carry.* In this condition, the highest accuracy (96.7%) is still given by *P* = 7, with 3 clusters, and the lower one (94.1%) is represented by *P* = 15, again with 3 clusters. The confusion matrix for these two configurations are shown in Figure 4, where the main difference is the reduced misclassification between the activities* walk* and* carry*, that are very similar to each other.

#### 4.2.4. Florence3D Dataset

The Florence3D dataset is composed of 9 activities and 10 people performing them multiple times, resulting in a total number of 215 activities. The proposed setting to evaluate this dataset is the leave-one-actor-out, which is equivalent to the “new-person” setting previously described. The minimum number of frames is 8, so the following sequence of clusters has been considered: *K* = [3,4, 5,6, 7,8]. A number of 15 joints are available for the skeleton, so only the first three schemes are included in the tests.

Table 8 shows the results obtained with different subsets of joints, where the best result for the proposed algorithm is given by *P* = 11, with 6 clusters. The choice of clusters does not significantly affect the performance, because the maximum reduction is 2%. In this dataset, the proposed approach does not achieve state-of-the-art accuracy (96.2%), given by Taha et al. [28]. However, all the algorithms that overcome the proposed one exhibit greater complexity because they consider Lie group representations [33, 34], or several machine learning algorithms, such as SVM combined to HMM, to recognize activities composed of atomic actions [28].

If different subsets of joints are considered, the performance decreases. In particular, considering only 7 joints and 3 clusters it is possible to reach a maximum accuracy of 82.1%, which is very similar to the one obtained by Seidenari et al. [44] who collected the dataset. By including all the available joints (15) in the algorithm processing, it is possible to achieve an accuracy which varies between 84.0% and 84.7%.

**Figure 5:**
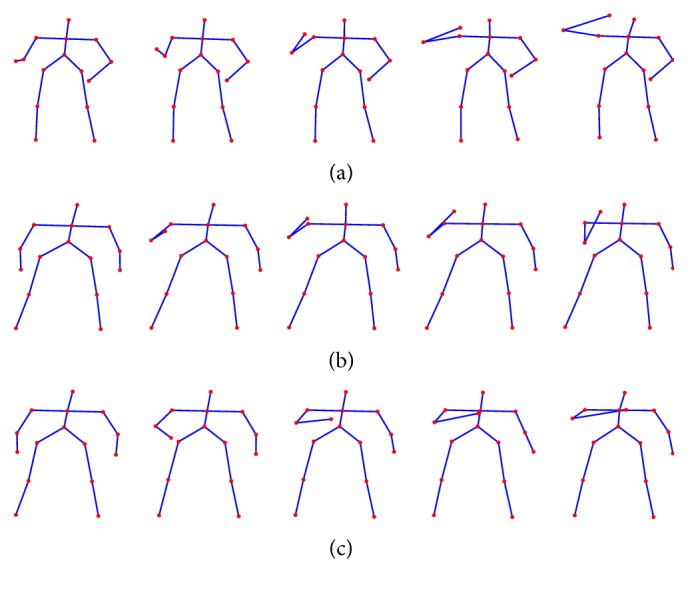
Sequences of frames representing the* drink from a bottle* (a),* answer phone* (b), and* read watch* (c) activities from Florence3D dataset.

The Florence3D dataset is challenging because of two reasons:

- High interclass similarity: some actions are very similar to each other, for example,* drink from a bottle*,* answer phone*, and* read watch*. As can be seen in Figure 5, all of them consist of an uprising arm movement to the mouth, hear, or head. This fact affects the performance because it is difficult to classify actions consisting of very similar skeleton movements.
- High intraclass variability: the same action is performed in different ways by the same subject, for example, using left, right, or both hands indifferently.

**Figure 6:**
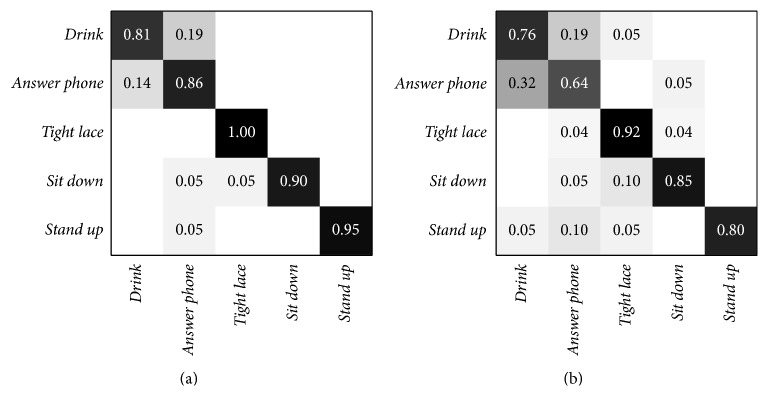
Confusion matrices of the Florence3D dataset with only AAL related activities. (a) Best accuracy confusion matrix, obtained with *P* = 11. (b) Worst accuracy confusion matrix, obtained with *P* = 7.

Limiting the analysis to AAL related activities only, the following ones can be selected:* drink*,* answer phone*,* tight lace*,* sit down*, and* stand up*. The algorithm reaches the highest accuracy (90.8%), using the “new-person” setting, with *P* = 11 joints and *K* = 3. The confusion matrix obtained in this condition is shown in Figure 6(a). On the other hand, the worst accuracy is 79.8% and it is obtained with *P* = 7 and *K* = 4. In Figure 6(b), the confusion matrix for this scenario is shown. In both situations, the main problem is given by the similarity of* drink* and* answer phone* activities.

#### 4.2.5. MSR Action3D Dataset

The MSR Action3D dataset is composed of 20 activities which are mainly gestures. Its evaluation is included for the sake of completeness in the comparison with other activity recognition algorithms, but the dataset does not contain AAL related activities. There is a big confusion in the literature about the validation tests to be used for MSR Action3D. Padilla-López et al. [49] summarized all the validation methods for this dataset and recommend using all the possible combinations of 5-5 subjects splitting or using LOAO. The former procedure consists of considering 252 combinations of 5 subjects for training and the remaining 5 for testing, while the latter is the leave-one-actor-out, equivalent to the “new-person” scheme previously introduced. This dataset is quite challenging, due to the presence of similar and complex gestures; hence the evaluation is performed by considering three subsets of 8 gestures each (AS1, AS2, and AS3 in Table 9) as suggested in [45]. Since the minimum number of frames constituting one sequence is 13, the following set of clusters has been considered: *K* = [3,5, 8,10,13]. All the combinations of 7, 11, 15, and 20 joints shown in Figure 2 are included in the experimental tests.

**Figure 7:**
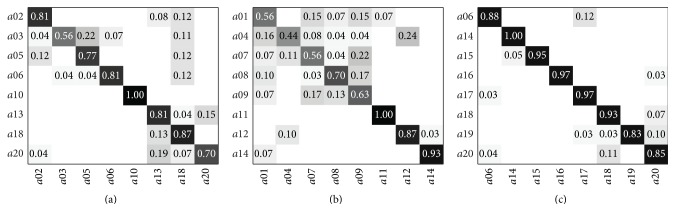
Confusion matrices of the MSR Action3D dataset obtained with *P* = 7 and the “new-person” test. (a) AS1, (b) AS2, and (c) AS3.

Considering the “new-person” scheme, the proposed algorithm tested separately on the three subsets of MSR Action3D reaches an average accuracy of 81.2% with *P* = 7 and *K* = 10. The confusion matrices for the three subsets are shown in Figure 7, where it is possible to notice that the algorithm struggles in the recognition of the AS2 subset (Figure 7(b)), mainly represented by drawing gestures. Better results are obtained processing the subset AS3 (Figure 7(c)), where lower recognition rates are shown for complex gestures:* golf swing* and* pickup* and* throw*.  Table 10 shows the results obtained including also other joints, which are slightly worse. A comparison with previous works validated using the “new-person” test is shown in Table 11. The proposed algorithm achieves results comparable with [50], which exploits an approach based on skeleton data. Chaaraoui et al. [15, 36] exploits more complex algorithms, considering the fusion of skeleton and depth data, or the evolutionary selection of the best set of joints.

## 5. Discussion

The proposed algorithm, despite its simplicity, is able to achieve and sometimes to overcome state-of-the-art performance, when applied to publicly available datasets. In particular, it is able to outperform some complex algorithms exploiting more than one classifier in KARD and CAD-60 datasets.

Limiting the analysis to AAL related activities only, the algorithm achieves interesting results in all the datasets. The group of 8 activities labeled as Actions in the KARD dataset is recognized with an accuracy greater than 98.7% in the considered experiments, and the group includes two similar activities, such as* phone call* and* drink*. The CAD-60 dataset contains only actions, so all the activities, considered within the proper location, have been included in the evaluation, resulting in global precision and recall of 93.9% and 93.5%, respectively. In the UTKinect dataset the performance improves considering only the AAL activities, and the same happens also in the Florence3D, having the highest accuracy close to 91%, even if some actions are very similar.

The MSR Action3D is a challenging dataset, mainly comprising gestures for human-computer interaction, and not actions. Many gestures, especially the ones included in AS2 subset, are very similar to each other. In order to improve the recognition accuracy, more complex features should be considered, including not only joints' relative positions but also their velocity. Many sequences contain noisy skeleton data: another approach to improving recognition accuracy could be the development of a method to discard noisy skeleton or to include depth-based features.

However, the AAL scenario raises some problems which have to be addressed. First of all, the algorithm exploits a multiclass SVM, which is a good classifier but it does not make easy to understand if an activity belongs or not to any of the training classes. In fact, in a real scenario, it is possible to have a sequence of frames that does not represent any activity of the training set: in this case, the SVM outputs the most likely class anyway, even if it does not make sense. Other machine learning algorithms, such as HMMs, distinguish among multiple classes using the maximum posterior probability. Gaglio et al. [29] proposed using a threshold on the output probability to detect unknown actions. Usually, SVMs do not provide output probabilities, but there exist some methods to extend SVM implementations and make them able to provide also this information [53, 54]. These techniques can be investigated to understand their applicability in the proposed scenario.

Another problem is segmentation of actions. In many datasets, the actions are represented as segmented sequences of frames, but in real applications the algorithm has to handle a continuous stream of frames and to segment actions by itself. Some solutions for segmentation have been proposed, but most of them are based on thresholds on movements, which can be highly data-dependent [46]. Also this aspect has to be further investigated, to have a system which is effectively applicable in a real AAL scenario.

## 6. Conclusions

In this work, a simple yet effective activity recognition algorithm has been proposed. It is based on skeleton data extracted from an RGBD sensor and it creates a feature vector representing the whole activity. It is able to overcome state-of-the-art results in two publicly available datasets, the KARD and CAD-60, outperforming more complex algorithms in many conditions. The algorithm has been tested also over other more challenging datasets, the UTKinect and Florence3D, where it is outperformed only by algorithms exploiting temporal alignment techniques, or a combination of several machine learning methods. The MSR Action3D is a complex dataset and the proposed algorithm struggles in the recognition of subsets constituted by similar gestures. However, this dataset does not contain any activity of interest for AAL scenarios.

Future works will concern the application of the activity recognition algorithm to AAL scenarios, by considering action segmentation, and the detection of unknown activities.

## Figures and Tables

**Table 1:** Activity sets grouping different and similar activities from KARD dataset.

Activity Set 1	Activity Set 2	Activity Set 3
*Horizontal arm wave *	* High arm wave *	* Draw tick *
*Two-hand wave *	* Side kick *	* Drink *
*Bend *	* Catch cap *	* Sit down *
*Phone call *	* Draw tick *	* Phone call *
*Stand up *	* Hand clap *	* Take umbrella *
*Forward kick *	* Forward kick *	* Toss paper *
*Draw X*	* Bend *	* High throw *
*Walk *	* Sit down *	* Horizontal arm wave *

**Table 2:** Accuracy (%) of the proposed algorithm compared to the other using KARD dataset with different Activity Sets and for different experiments.

	Activity Set 1			Activity Set 2			Activity Set 3		
	A	B	C	A	B	C	A	B	C
Gaglio et al. [29]	95.1	99.1	93.0	89.9	94.9	90.1	84.2	89.5	81.7
Proposed (*P* = 7)	98.2	98.4	98.1	99.7	100	99.7	90.2	95.0	91.3
Proposed (*P* = 11)	98.0	99.0	97.7	99.8	100	99.6	91.6	95.8	93.3
Proposed (*P* = 15)	97.5	98.8	97.6	99.5	100	99.6	91	95.1	93.2

**Table 3:** Accuracy (%) of the proposed algorithm compared to the other using KARD dataset, with dataset split in Gestures and Actions, for different experiments.

	Gestures			Actions		
	A	B	C	A	B	C
Gaglio et al. [29]	86.5	93.0	86.7	92.5	95.0	90.1
Proposed (*P* = 7)	89.9	93.5	92.5	99.1	99.6	99.4
Proposed (*P* = 11)	89.9	95.9	93.7	99.0	99.9	99.1
Proposed (*P* = 15)	87.4	93.6	92.8	98.7	99.5	99.3

**Table 4:** Precision (%) and recall (%) of the proposed algorithm compared to the other, using the whole KARD dataset and “new-person” setting.

Algorithm	Precision	Recall
Gaglio et al. [29]	84.8	84.5
Proposed (*P* = 7)	94.0	93.7
Proposed (*P* = 11)	95.1	95.0
Proposed (*P* = 15)	95.0	94.8

**Table 5:** Precision (%) and recall (%) of the proposed algorithm, in the different environments of CAD-60, with *P* = 11 and *K* = 25.

Location	Activity	“New-person”	
		Precision	Recall
Bathroom	*Brushing teeth *	88.9	100
	*Rinsing mouth *	92.3	100
	*Wearing contact lens *	100	79.2
	Average	93.7	93.1

Bedroom	*Talking on phone *	91.7	91.7
	*Drinking water *	91.3	87.5
	*Opening pill container *	96.0	100
	Average	93.0	93.1

Kitchen	*Cooking-chopping *	85.7	100
	*Cooking-stirring *	100	79.1
	*Drinking water *	96.0	100
	*Opening pill container *	100	100
	Average	95.4	94.8

Living room	*Talking on phone *	87.5	87.5
	*Drinking water *	87.5	87.5
	*Talking on couch *	88.9	100
	*Relaxing on couch *	100	87.5
	Average	91.0	90.6

Office	*Talking on phone *	100	87.5
	*Writing on whiteboard *	100	95.8
	*Drinking water *	85.7	100
	*Working on computer *	100	100
	Average	96.4	95.8

	Global average	93.9	93.5

**Table 6:** Global precision (%) and recall (%) of the proposed algorithm for CAD-60 dataset and “new-person” setting, with different subsets of joints, compared to other works.

Algorithm	Precision	Recall
Sung et al. [43]	67.9	55.5
Gaglio et al. [29]	77.3	76.7
Proposed (*P* = 15)	87.9	86.7
Faria et al. [47]	91.1	91.9
Parisi et al. [48]	91.9	90.2
Proposed (*P* = 7)	92.7	91.5
Shan and Akella [46]	93.8	94.5
Proposed (*P* = 11)	93.9	93.5

**Table 7:** Global accuracy (%) of the proposed algorithm for UTKinect dataset and LOOCV setting, with different subsets of joints, compared to other works.

Algorithm	Accuracy
Xia et al. [26]	90.9
Theodorakopoulos et al. [30]	90.95
Ding et al. [31]	91.5
Zhu et al. [17]	91.9
Jiang et al. [35]	91.9
Gan and Chen [24]	92.0
Liu et al. [22]	92.0
Proposed (*P* = 15)	93.1
Proposed (*P* = 11)	94.2
Proposed (*P* = 19)	94.3
Anirudh et al. [34]	94.9
Proposed (*P* = 7)	95.1
Vemulapalli et al. [33]	97.1

**Table 8:** Global accuracy (%) of the proposed algorithm for Florence3D dataset and “new-person” setting, with different subsets of joints, compared to other works.

Algorithm	Accuracy
Seidenari et al. [44]	82.0
Proposed (*P* = 7)	82.1
Proposed (*P* = 15)	84.7
Proposed (*P* = 11)	86.1
Anirudh et al. [34]	89.7
Vemulapalli et al. [33]	90.9
Taha et al. [28]	96.2

**Table 9:** Three subsets of gestures from MSR Action3D dataset.

AS1	AS2	AS3
[*a*02] *Horizontal arm wave*	[*a*01] *High arm wave*	[*a*06] *High throw*
[*a*03] *Hammer*	[*a*04] *Hand catch*	[*a*14] *Forward kick*
[*a*05] *Forward punch*	[*a*07] *Draw X*	[*a*15] *Side kick*
[*a*06] *High throw*	[*a*08] *Draw tick*	[*a*16] *Jogging*
[*a*10] *Hand clap*	[*a*09] *Draw circle*	[*a*17] *Tennis swing*
[*a*13] *Bend*	[*a*11] *Two-hand wave*	[*a*18] *Tennis serve*
[*a*18] *Tennis serve*	[*a*12] *Side boxing*	[*a*19] *Golf swing*
[*a*20] *Pickup and throw*	[*a*14] *Forward kick*	[*a*20] *Pickup and throw*

**Table 10:** Accuracy (%) of the proposed algorithm for MSR Action3D dataset and “new-person” setting, with different subsets of joints and different subsets of activities.

Algorithm	AS1	AS2	AS3	Avg.
Proposed (*P* = 15)	78.5	68.8	92.8	80.0
Proposed (*P* = 20)	79.0	70.2	91.9	80.4
Proposed (*P* = 11)	77.6	73.7	91.4	80.9
Proposed (*P* = 7)	79.5	71.9	92.3	81.2

**Table 11:** Average accuracy (%) of the proposed algorithm for MSR Action3D dataset and “new-person” setting, compared to other works.

Algorithm	Accuracy
Çeliktutan et al. (2015) [51]	72.9
Azary and Savakis (2013) [52]	78.5
Proposed (*P* = 7)	81.2
Azary and Savakis (2012) [50]	83.9
Chaaraoui et al. (2013) [15]	90.6
Chaaraoui et al. (2014) [36]	93.5
